# High-Amylose Maize, Potato, and Butyrylated Starch Modulate Large Intestinal Fermentation, Microbial Composition, and Oncogenic miRNA Expression in Rats Fed A High-Protein Meat Diet

**DOI:** 10.3390/ijms20092137

**Published:** 2019-04-30

**Authors:** Tina S. Nielsen, Zach Bendiks, Bo Thomsen, Matthew E. Wright, Peter K. Theil, Benjamin L. Scherer, Maria L. Marco

**Affiliations:** 1 Department of Animal Science, Aarhus University, DK-8830 Tjele, Denmark; Peter.Theil@anis.au.dk; 2 Department of Food Science & Technology, University of California, Davis, CA 95616, USA; zabendiks@ucdavis.edu (Z.B.); merwright@ucdavis.edu (M.E.W.); mmarco@ucdavis.edu (M.L.M.); 3 Department of Molecular Biology & Genetics, Aarhus University, DK-8000 Aarhus C, Denmark; Bo.Thomsen@mbg.au.dk; 4 Commenwealth Scientific and Industrial Research Organisation (CSIRO), Biosecurity & Health, Adelaide BC, SA 5000, Australia; Ben.Scherer@csiro.au

**Keywords:** butyrate, resistant starch, colon, short-chain fatty acids, butyrate, microbiome, micro-RNA expression, DNA-adduct

## Abstract

High red meat intake is associated with the risk of colorectal cancer (CRC), whereas dietary fibers, such as resistant starch (RS) seemed to protect against CRC. The aim of this study was to determine whether high-amylose potato starch (HAPS), high-amylose maize starch (HAMS), and butyrylated high-amylose maize starch (HAMSB)—produced by an organocatalytic route—could oppose the negative effects of a high-protein meat diet (HPM), in terms of fermentation pattern, cecal microbial composition, and colonic biomarkers of CRC. Rats were fed a HPM diet or an HPM diet where 10% of the maize starch was substituted with either HAPS, HAMS, or HAMSB, for 4 weeks. Feces, cecum digesta, and colonic tissue were obtained for biochemical, microbial, gene expression (oncogenic microRNA), and immuno-histochemical (O^6^-methyl-2-deoxyguanosine (O^6^MeG) adduct) analysis. The HAMS and HAMSB diets shifted the fecal fermentation pattern from protein towards carbohydrate metabolism. The HAMSB diet also substantially increased fecal butyrate concentration and the pool, compared with the other diets. All three RS treatments altered the cecal microbial composition in a diet specific manner. HAPS and HAMSB showed CRC preventive effects, based on the reduced colonic oncogenic miR17-92 cluster miRNA expression, but there was no significant diet-induced differences in the colonic O^6^MeG adduct levels. Overall, HAMSB consumption showed the most potential for limiting the negative effects of a high-meat diet.

## 1. Introduction

Intake of red and processed meat is positively associated with the risk of colorectal cancer (CRC) [1]. In contrast, human studies indicate that diets high in dietary fibers (DF), such as resistant starch (RS) reduce the CRC risk [1]. Evidence of a protective effect of RS against CRC is supported by some [2,3] but not all [4,5] studies using rodent models. Several mechanisms to explain the association between red meat consumption and CRC risk have been suggested. These mechanisms implicate metabolites produced by the gut microbiota from undigested dietary proteins, heme iron, heterocyclic aromatic amines, synthesized upon cooking and an endogenous formation of *N*-nitroso compounds, which might all negatively impact the colonic epithelial cell physiology [6]. In particular, *N*-nitroso compounds are alkylating agents that generate DNA-adducts in colonocytes [7], one of them being the pro-mutagenic O^6^-methyl-2-deoxyguanosine (O^6^MeG) adduct that is increased in both murine [8] and human [9] colonocytes, following consumption of a high red meat diet. More recently, it has also been suggested that high quantities of red meat intake can increase the expression of oncogenic microRNA’s (miRNA), such as the miR17-92 cluster (miR17, miR18a, miR19a, miR20a, miR19b and miR92a) and miR21 in human rectal epithelial cells [10]. MicroRNAs are small (18-25 nucleotides) non-coding RNAs that down-regulate gene expression [11], and alterations in colonic miRNA expression are strong predictive biomarkers of future neoplasia in animal models [12]. Production of the short-chain fatty acids (SCFA), acetate, propionate, and butyrate, via bacterial fermentation in the large intestine [13] might, on the other hand, provide an important link between intake of DF and a reduced CRC risk. Butyrate especially appears to have a crucial role, as it is the preferred metabolic substrate for colonocytes. Butyrate also has its strong anti-tumorigenic properties [14] and was shown to modulate oncogenic miRNA expression [15]. 

Animal studies have shown that the negative effects of diets with high levels of red meat, on colo-rectal health, can be attenuated by the high-amylose maize starch (HAMS) that is high in Type 2 RS. Type 2 RS is not subject to rapid enzymatic digestion in the small intestine, due to starch granule conformation [8,16,17,18] and is digested at a much slower rate than thee non-resistant starch. HAMS might function by shifting the colonic microbial metabolism away from protein, towards the metabolism of carbohydrates [8]. Toden et al. [16] reported that the dose-dependent reduction in red meat-induced colonic DNA damage by HAMS, correlated more highly with cecal butyrate than with other SCFAs in rats, consistent with butyrate’s proposed role in promoting colonic integrity. HAMS can also be acylated with butyrate to produce butyrylated HAMS (HAMSB). Intestinal bacteria can release butyrate from HAMSB, thereby, delivering butyrate directly to colonocytes. This can also lead to increased fecal butyrate, compared with the standard HAMS [19,20]. In humans, including HAMSB in a diet enriched in red meat, altered fecal microbial composition and prevented rectal O^6^MeG DNA-adduct formation [9]. Dose-dependent positive effects of HAMSB on CRC markers was also found in rats [21]. Abell et al. reported different fecal microbial profiles between rats receiving low- or high-amylose maize starch and HAMSB [22]. These results corresponded well with recent findings showing that the inclusion of HAMSB in a high red meat diet, changed the urinary excretion of potentially harmful metabolites derived from the gut microbiota in rats [23]. 

HAMS has gained substantial attention in relation to its CRC protective effects. However, the potential of starch from other high-amylose hybrids of plants, such as high-amylose potato starch (HAPS) [24], a starch that is also relatively high in RS, has not been investigated previously. The aim of this study was to determine whether HAPS, HAMS, and HAMSB, the latter synthesized by a simple organocatalytic route [25,26], could oppose the negative effects of a high red meat diet, in terms of microbial metabolism in the large intestine, intestinal microbial composition, and biomarkers of CRC in rats.

## 2. Results

### 2.1. BW, Feed Intake, and Fermentation Products

Diet did not induce differences in body weight at the completion of the study (360 g, SEM = 14.4; *p* = 1.0). The feed intake was not affected by diet during week 1 (on average 17.0 g/rat/d, SEM = 1.8; *p* = 0.30) when registered on cage level (n = 2ߝ3 per cage) and also not during week 3 (15.9 g/rat/d, SEM = 2.0; *p* = 0.64) where feed intake was registered at the individual level in metabolism cages. 

The concentration and pool of carbohydrate and protein fermentation products in the cecum digesta and feces are presented in Table 1. The amount of cecum digesta was 35% higher in rats fed the HAMS and HAMSB diet, compared with the two other diets. The HAMSB diet lowered the cecal acetate concentration, whereas propionate concentration and pool was unaffected by the diet. Rats fed the control HPM diet showed the lowest cecal concentration (12 µmol/g) and the lowest pool (21 µmol) of butyrate, whereas the HAMSB diet resulted in the highest concentration (24 µmol/g) and pool (62 µmol) of butyrate. This was a 58% increase in the butyrate concentration and 72% increase in the butyrate pool, compared to the native HAMS. HAPS resulted in the second highest concentration of cecal butyrate (19 µmol/g). The HAPS and HAMS diets increased the cecal total organic acid concentration by 21%, compared with the HPM diet, the HAMSB diet being intermediate. The pool of cecal total organic acid was largest, following the HAMS and HAMSB diets (229 µmol/g on average), intermediate following the HAPS diet (181 µmol/g), and lowest after the HPM diet (139 µmol/g).

For the concentration and pool of the protein fermentation products, branched-chain fatty acids (sum of iso-butyrate, iso-valerate; (BCFA)), p-cresol, phenol, and indole, there was no effect of including HAPS in the HPM diet (Table 1). In contrast, HAMS and HAMSB reduced the cecal concentration of BCFA by 32% and 55%, respectively, compared with the HPM diet. Including HAMS and HAMSB in the HPM diet also reduced the *p*-cresol concentration by 51% and HAMS reduced both phenol concentration and pool by 80% and 71%, respectively. Cecal indole concentration was affected by the diet to a minor degree (*p* < 0.01), with the HAMS diet resulting in the lowest (14 µmol/g) and the HAPS and HPM diets in the highest concentration (20 µmol/g on average). 

The fecal output (g wet weight/rat/dg) was 88% higher in HAMSB than in HPM and HAPS-fed rats with HAMS-fed rats were intermediate. Both acetate and propionate concentrations and pools in feces were highly dependent on diet (*p* < 0.001). For butyrate, the HAMSB diet increased the fecal concentration more than 8 times and the fecal pool more than 27 times, relative to the other diets. The HAMS and HAMSB diets increased fecal total organic acid concentration 2.5 times (average 127 µmol/g) and the fecal total organic acid pool 4 times (average 498 µmol), compared with the HPM and HAPS treatments. The BCFA concentration in feces was lowest, following the HAMSB diet (0.5 µmol/g) and the highest for the HAPS-fed rats (1.4 µmol/g). Inclusion of HAMS in the HPM diet reduced the fecal p-cresol concentration by 80% and the pool by 72%, whereas the HAPS and HAMSB diets resulted in a minor reduction (34% on average), compared to the HPM diet. The fecal indole concentration was reduced substantially, following the HAMS diet relative to the HPM diet, with less substantial reductions also observed for the HAMSB and HAPS diets. Only HAMS resulted in a lower fecal indole pool, compared with the other treatments. 

### 2.2. The Gut Microbiota is Influenced by the RS Type

**Figure 1:**
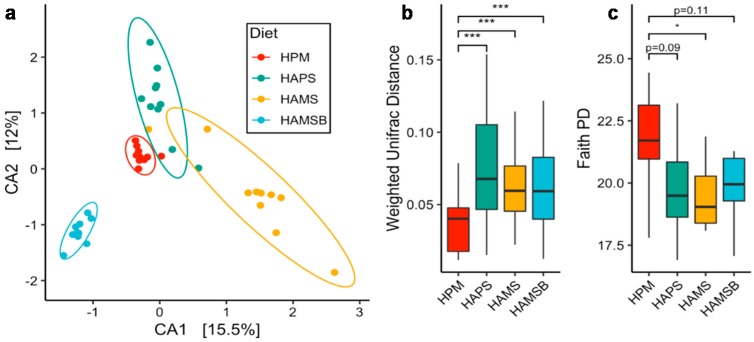
Resistant starch changes the composition of the rat cecal microbiota. (**a**) Canonical correspondence analysis of the weighted UniFrac distance metric. Each point represents an individual rat cecal community. (**b**) Boxplots of weighted UniFrac distances and (**c**) Faith’s Phylogenetic Diversity (PD). HPM—high protein meat, HAPS—HPM + high amylose potato starch, HAMS—HPM + high amylose maize starch, HAMSB—HPM + butyrylated high amylose maize starch. ANOVA followed by pairwise Tukey HSD was performed for quantifying the statistical differences in alpha and beta diversity (*n* = 10 rats per treatment group). * *p* < 0.5, *** *p* < 0.001.

Ordination of the gut communities showed that rat cecal microbiota formed distinct clusters according to the diet intervention (Figure 1a), and the weighted UniFrac distances between the HPM and the HAPS, HAMS, and HAMSB groups were significantly greater than the distance within the HPM group (Figure 1b). This showed that gut community composition in rats fed HAPS, HAMS, and HAMSB, was distinct from rats consuming HPM. Associated with this shift in microbial composition, was a significant reduction in the phylogenetic diversity in HAMS, compared to the HPM, while HAPS and HAMSB trended toward a lower diversity as well (Figure 1c and Supplementary Figure S1). Together, these results showed that the three RS diets tested were effective at modulating gut microbial composition and lowering the alpha diversity, although to varying degrees.

**Figure 2:**
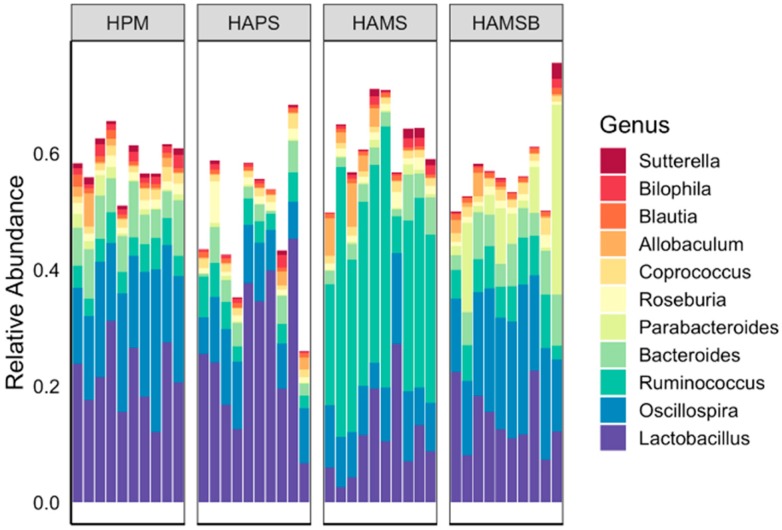
Relative abundance of bacterial genera in rat cecal contents. Each bar represents the bacterial composition in individual cecal samples. Genera constituting ≥ 0.01 of total bacteria present are shown.

**Figure 3:**
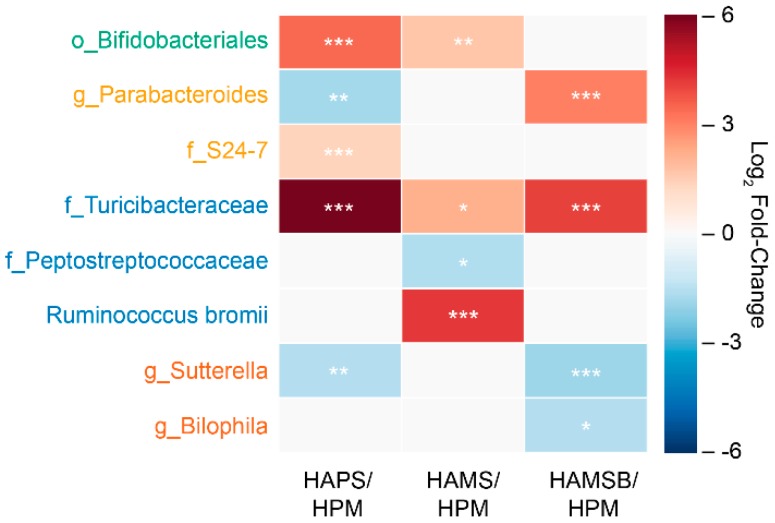
Bacterial taxa found in significantly different proportions in rats fed resistant starch (RS) diets, compared to the rats fed the HPM diet. The log_2_-transformed, fold-changes of the relative abundance of bacteria in the RS diets, compared to the HPM diet are shown. Only taxa that were significantly changed in an RS diet compared to HPM are shown. A log_2_ fold-change greater or equal to 1.0 and a *p*-value < 0.05, determined by the Wald test in DESeq2, was necessary to be considered significant. The scale represents the log_2_-fold change, and the heatmap is annotated such that * *p* < 0.05, ** *p* < 0.01, *** *p* < 0.001. The taxon names are colored according to the phyla as follows—Actinobacteria (green), Bacteroidetes (gold), Firmicutes (blue), and Proteobacteria (orange).

The cecal microbiota contained similar bacterial taxa among all diet groups and were enriched in members of the *Ruminococcus, Oscillospira, Lactobacillus*, and *Bacteroides* genera (between 20 and 70% of all bacteria detected) (Figure 2). However, the proportions of certain bacteria varied between rats, in a diet-dependent manner. Rats receiving the HAMS diet contained significantly higher proportions of bacteria, similar to *Ruminococcus bromii* (*R. bromii*) (19% to 46% range of total; average of 18-fold increase) (Figure 3 and Table 2). HAMS-fed rats also harbored elevated numbers of the low-abundance taxa (0.06% to 1% range of total) in the Turicibacteriaceae family (4.5-fold increase) and Bifidobacteriales order (3.2-fold increase) and lower numbers of bacteria in the Peptostreptococcaceae family (2.8-fold decrease), compared to rats fed the HPM diet (Figure 3 and Table 2).

Although HAMSB was synthesized from HAMS, the intestinal microbiota differed between the rats fed these two diets. Most notably, the levels of *R. bromii* were only increased in rats consuming HAMS and not the butyrylated form (Figure 3 and Table 2). The proportions of Bifidobacteriales were also unchanged on the HAMSB diet, compared to the HPM control diet (Figure 3 and Table 2). Both HAMS- and HAMSB-fed rats contained higher proportions of *Turicibactereaceae* relative to the HPM controls, but only the HAMS and not HAMSB resulted in significantly lower proportions of *Peptostreptococcaceae.* Instead, HAMSB resulted in significant reductions of the Proteobacteria species *Sutterella* (0.09–2.6% range of total; 3.5-fold change) and *Bilophila* (0.04–1.6% range of total; 2.8-fold change). The HAMSB diet also caused increases in *Parabacteroides*, a genus in the Bacteroidetes phylum, compared to the rats fed either HPM or HAMS (0.6–32% range of total; 8.4-fold increase) (Table 2).

Lastly, HAPS-fed rats showed an increase in bacteria in the Bifidobacteriales order, compared to the HPM diet (up to 10.2% of total; 11-fold increase) (Figure 3 and Table 2). *Lactobacillaceae* were also found in high relative abundance overall in rats receiving the HAPS diet (on average, 26% of total bacteria present), although the increase did not reach statistical significance, compared to the HPM control treatment (Table 2). HAPS also resulted in higher proportions of *Turicibactereaceae* (up to 3.4% of total; 69-fold increase) as well as the S_24-7 Bacteroidetes family, (1.2–9.2% range; 2.6-fold change) (Figure 3 and Table 2). The levels of *Parabacteroides* and *Sutterella* were reduced (–3.2-fold and –2.8-fold, respectively) in rats fed HAPS, compared to those given HPM. 

### 2.3. Correlations between Microbial Abundance and Cecal Metabolites

**Figure 4:**
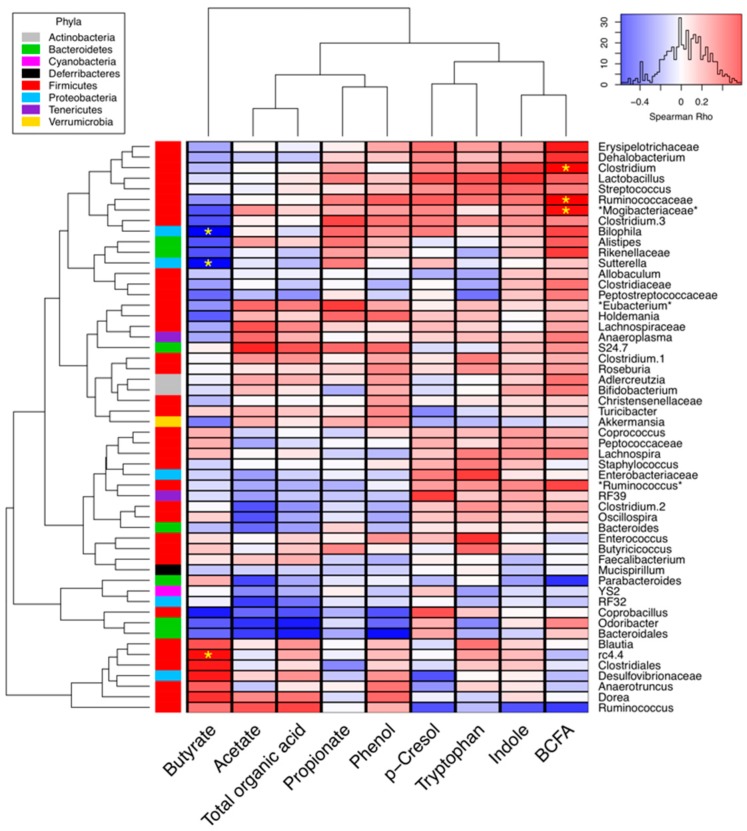
Correlation heatmap between the cecal microbiota and cecal metabolite concentrations. Taxa detected in less than 500 total DNA sequences were removed from the analysis. Blue and red cells in the heatmap represent the negative and positive Spearman rho values, respectively, and yellow asterisks denote the significant correlations (false discovery ratecorrected *p* < 0.05). The colored vertical bar (left side) shows the corresponding phylum. Predicted annotations from the Greengenes database have asterisks before and after their taxonomic label. Taxa and metabolite dendrograms are based on Euclidean distances between features.

To further explore the relationship between cecal metabolites and microbiota, the cecal metabolite concentrations were compared to the proportions of microbial taxa (Figure 4). BCFA and butyrate concentrations were significantly correlated with microbial abundance, based on Spearman rho values. *Clostridium, Ruminococcaceae*, and bacteria in a putative family known as *Mogibacteriaceae* were positively correlated with the BCFA concentrations (*p* < 0.05). Only the candidate genus rc4.4 in the Firmicutes phylum was significantly positively associated with butyrate concentrations, where, as two *Proteobacteria* genera, *Bilophila* and *Sutterella*, were negatively correlated with the cecal butyrate concentration.

### 2.4. Colonic Oncogenic miRNA Expression

**Figure 5:**
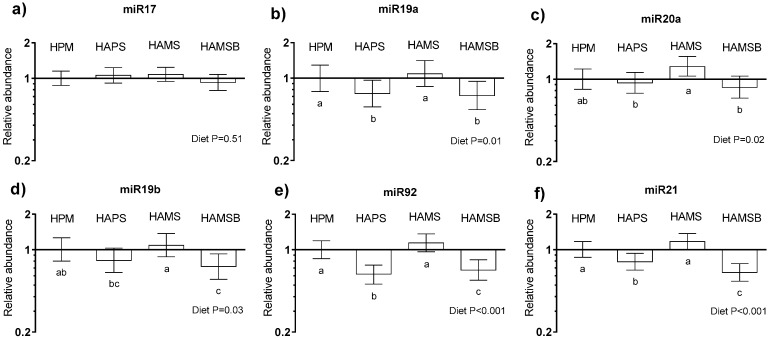
Relative abundance of miRNA’s in mucosal scrapings of the distal colon of rats fed the HPM, HAPS, HAMS, or the HAMSB diet (*n* = 10/group). (**a**) miR17, (**b**) miR19a, (**c**) miR20a, (**d**) miR19b, (**e**) miR92a, and (**f**) miR21. Data are presented as the means ± 95% confidence intervals relative to the abundance in the HPM-fed rats (=1) and are normalized against the beta-actin as the reference gene. Note that the scale on the Y-axis is logarithmic and that the units are arbitrary. Statistically significant (*p* < 0.05) effects between diets are indicated with different letters (a, b, c).

Of the potential reference genes to be used for normalization of the miRNA abundance data, beta-actin was not regulated by dietary treatment (*p* = 0.86), in contrast to hypoxanthine phosphoribosyltransferase 1 (HPRT-1) (*p* = 0.01). Therefore, only beta-actin was considered suitable and included in the analysis as a reference gene. The miR17-92 cluster miRNA levels were examined in mucosal scrapings of the colon, as these and miR21 were shown to be elevated in CRC [27], following high red-meat-intakes [10]. The relative abundance of the miR17-92 cluster (miR17, miR19a, miR20a, miR19b, and miR92a) and of miR21 is shown in Figure 5a–f. The miRNA abundance following the HAPS, HAMS, and HAMSB diets was expressed relative to the HPM diet (=1). The diet showed a significant effect on four of the five miR17-92 cluster (not miR17) levels. Relative to the HPM diet, the HAMS diet did not affect the miR17-92 cluster levels. The expression of miR19a was reduced by 25% in the HAPS and the HAMSB-fed rats, compared to the HPM-fed rats. The HAMSB diet resulted in 30% lower levels of miR19b and miR92a, compared with the HPM and HAMS diets, and the miR92 levels was 40% lower, following the HAPS, compared with the HPM and HAMS diets. HAMS resulted in the highest levels of miR20a, while the HAPS and HAMSB diets resulted in the lowest levels, with the HPM diet resulting in intermediate levels. The relative abundance of the miR21 was 40% lower for the HAMSB-fed rats and 20% lower for the HAPS-fed rats than for the HPM and the HAMS-fed rats. 

### 2.5. O^6^MeG Adduct Formation 

**Figure 6:**
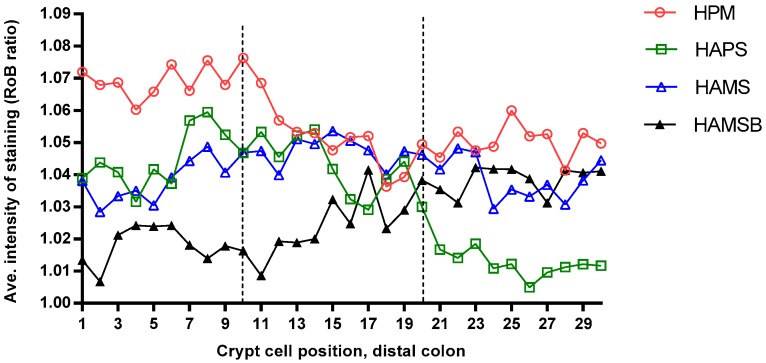
Average distribution of O^6^MeG DNA-adduct formation in the distal colon of rats receiving a high protein meat (HPM), the HPM diet plus 10% high-amylose maize starch (HAMS), the HPM plus 10% high-amylose potato starch (HAPS), or the HPM plus 10% butyrylated HAMS (HAMSB), for four weeks. The highest cell number on the X-axis represents the top of the crypt, with the delineations for the lower-, middle-, and the upper-crypt-thirds. Data are presented as red color over blue color ratio (RoB); a higher value represents a higher intensity of positive staining in the cell nucleus.

There was no statistical significant difference in the average intensity of staining (RoB ratio) between diets in the lower (*p* = 0.47), mid (*p* = 0.82), and upper (*p* = 0.82) thirds of the distal colonic crypts. However, the staining intensity was numerically highest for the rats fed the HPM diet and lowest for the HAMSB-fed rats in the lower third of the crypts, whereas in the upper third of the crypt, the HAPS-diet resulted in the numerically lowest staining intensity (Figure 6). 

## 3. Discussion

Both animal and human data strongly indicate that dietary RS in the form of HAMS or butyrylated HAMS, increase the level of beneficial cecal-colonic fermentation products and affect the CRC biomarkers, such as colonic DNA damage, following a red meat diet in a positive direction [8,9,16,17,18,28]. The aim of the present study was to evaluate RS as HAPS, HAMS, or HAMSB, the latter synthesized through a different route than the HAMSB previously applied in studies, in relation to effects on the large intestinal fermentation pattern, microbial composition, and colonic biomarkers of CRC, in rats fed a high red-meat-diet. Our HAPS was a non-genetically modified potato starch, currently under investigation for its functionality and not yet commercially available. In contrast to the HAMSB previously used in rodent and human studies [20,29], the HAMSB applied here was produced by a solvent-free, operationally simple, and environmentally benign methodology [25], resulting in a HAMSB, with a larger amount of butyrate attached to the starch polymer (degree of substitution; DS = 0.5). Our results showed that the fecal butyrate pool, used as an indicator of the distal colonic butyrate pool, was 16-fold larger after the intake of HAMSB, relative to HAMS. This was substantially more than the approximately 4-fold increase in the distal colonic butyrate pool previously reported after a 10% HAMSB (DS~0.23) intake in rats [29]. Additionally, in absolute amounts (µmol), our HAMSB seemed much more effective at delivering butyrate to the distal part of the colon [29], which is the most frequent site for CRC to develop [30]. 

As expected, the cecal and fecal fermentation pattern was highly dependent on dietary treatment. The lower amount of cecum digesta in HAPS, compared to the HAMS and HAMSB-fed rats, indicated that more of the HAPS was digested in the small intestine and less carbohydrate, therefore, was available for fermentation in the cecum and the colon, resulting in lower concentrations and pools of carbohydrate fermentation products. In general, small intestinal starch degradability was inversely related to the amylose content, due to the decreased enzyme accessibility [31]. Although both HAPS and HAMS are considered high-amylose varieties, amylose only constituted 42% of the dry matter in HAPS, versus 80% in HAMS [24], and HAPS also exhibited a lower content of RS than HAMS (25% versus 40% of the dry matter, respectively) [24]. This, in part, might explain the difference in cecal and fecal outputs. The HAPS diet only induced minor changes in the cecal concentrations and the pool of carbohydrate and protein fermentation products relative to the HAMS and HAMSB diets, suggesting a higher GI tract digestion in the host, rather than through the colonic microbial action.

The three RS-containing diets tested in this study, promoted changed to a cecal bacterial composition. All three RS-based diets were effective at shifting the intestinal bacterial community composition away from the control diet, as indicated by examining the beta-diversity with the Unifrac distance metric. A factor contributing to those differences was the reduction in the bacterial phylogenetic diversity (alpha-diversity), when RS was included in the diet. This reduction in alpha-diversity strongly indicated that RS selectively enriched members of the intestinal microbiota able to metabolize the RS or its breakdown products. This finding was in agreement with our prior studies, wherein reductions in alpha-diversity were found in humans [32] and rodents [33], following RS consumption. Moreover, our results were consistent with previous reports showing broad categorical changes in intestinal microbiota composition, depending on the starch type [34]. Consistent between studies was the enrichment of *R. bromii* with HAMS, and Parabacteroides with HAMSB diets [22,35]. A prior investigation in rats treated with the chemical carcinogen azoxymethane, also showed that the HAMS consumption enriched *R. bromii* whereas HAMSB enriched *Lactobacillus gasseri* and *Parabecteroides distasonis* [22]. Most notably was the higher proportions of *R. bromii*, according to both total presence (mean relative abundance of 27%) and increase, compared to the HPM control group (18-fold increase) in the study performed here. This result was consistent with studies showing a pivotal role of this species in the RS metabolism [36]. Remarkably, *R. bromii* levels did not reach similarly high proportions in rats fed with the HAMSB, and instead, a broader range of Firmicutes were enriched, along with some Bacteroidetes taxa. Since *R. bromii* might be one of the relatively few number of organisms responsible for the major fraction of butyrate production [37], these results could indicate that butyrate delivery by HAMSB had a negative feedback on the production of butyrate from the bacteria present. Interestingly, the family *Turicibacteraceae*, which was present at very low proportions in the control diet (0.01%), was enriched in all diets supplemented with RS, although the overall abundance was still relatively low (0.0024–0.007 relative abundance). Whether *Turicibacteraceae* are capable of contributing critical metabolic effects, when present as minor members of the gut microbial communities, remains to be elucidated, but our findings were consistent with previous reports [38,39]. 

Our findings showed that the relative abundance of *Bilophila* and *Sutterella*, was strongly negatively correlated with cecal butyrate concentrations. These genera were Gram-negative anaerobes that might have putative roles in inflammatory bowel disease and colon cancer in humans [40]. When human gut-derived communities were used to ferment whole grains in vitro, *Bilophila* were also negatively correlated with butyrate production, but positively correlated with iso-valerate and ammonia concentrations [41]. Reductions in the proportions of *Bilophila* in the RS diets were consistent with prior reports based on animal studies [42,43]. These observations suggest that RS shifts the metabolic potential of the gut microbiome to favor microbes capable of butyrate production, while microbes contributing deleterious pathways, such as hydrogen sulfide production, were suppressed.

Expression profiles of miRNAs differ along the gastrointestinal tract [44] and the miR17-92 cluster miRNAs and miR21 are commonly over-expressed in CRCs [27,45], where they might promote proliferation and angiogenesis, inhibit differentiation, and sustain cell survival [46]. Humphreys et al. [10] showed that the miR17-92 cluster expression was elevated in rectal mucosa of humans consuming a high red-meat-diet, whereas intake of HAMSB restored miR17-92 (but not miR21) to the baseline levels. A positive effect that might be ascribed to the increased level of butyrate in the distal colon, following the HAMSB intake [10], since butyrate has been shown to modulate miRNA expression in colorectal cancer cells in vitro and significantly decrease the miR17-92 cluster expression [15]. In line with these results we find that HAMSB lowered the expression of several colonic miR17-92 cluster and miR21 miRNAs, compared to the HPM diet. However, although inclusion of HAPS in the HPM diet did not change the fecal fermentation pattern to the same extent or increase fecal butyrate as much as the HAMS or the HAMSB diet did, the profile of colonic miR17-92 cluster expression, following HAPS intake, was quite similar to the HAMSB (Figure 5). This might indicate that HAPS opposed the negative effects of an HPM diet, for some miR17-92 cluster miRNAs and miR21 miRNAs, via mechanisms independent of, for example, butyrate.

The DNA-adduct O^6^-methyl-2-deoxyguanosine (O^6^MeG) is a known mutagenic lesion in the colonocytes of both animals and humans that under experimental conditions can be induced by exposure to the alkylating agent azoxymethane (AOM) or by intake of a high red-meat-diet [8,9,47]. If O^6^MeG adducts are left unrepaired, it can lead to mutations in the *K-ras* gene, a mechanism of human oncogene activation and tumor suppressor inactivation [48]. To our knowledge, the present study is the first to report dietary (red meat)-induced colonic O^6^MeG adducts in rats and the effects of including dietary RS on this O^6^MeG adduct level. In contrast to the results from a mouse and a human study [8,9], we were not able to significantly increase the level of distal colonic O^6^MeG adducts, through feeding an HPM diet, as compared with the RS-enriched diets. Our HPM diet did result in the highest and the HAMSB diet in the lowest numerical levels of O^6^MeG adducts in the lower third of the crypts, but overall the span in O^6^MeG staining intensity was narrow and the variation was large, compared to the differences that could be obtained between the AOM-treated and untreated rats [47]. Distal colonic O^6^MeG adducts have been positively correlated to the fecal levels of the protein fermentation product *p*-cresol and negatively correlated to fecal butyrate [8]. Our data also suggested a negative relationship between the O^6^MeG adducts and the fecal butyrate level, at least in the lower third of the crypt, whereas, fecal *p*-cresol did not appear to be associated with the O^6^MeG adduct level. 

In summary, our findings showed that HAMS and HAMSB, the latter produced by using an organocatalytic route, had clear capabilities to shift the fecal fermentation pattern in a beneficial direction, for CRC prevention, compared to an HPM diet. All three RS treatments profoundly altered the cecal microbial composition in a diet-specific manner and the HAPS and HAMSB treatments showed CRC preventive effects, based on colonic oncogenic miR17-92 cluster miRNA expression. Overall, the HAMSB consumption showed most potential for attenuating the negative effects of a diet high in red meat.

## 4. Materials and Methods 

### 4.1. Diets

A semisynthetic high-protein, high-fat, control diet (high-protein meat—HPM) was formulated, based on the AIN93G diet. Red meat (minced, cooked beef) was the sole source of protein and fat in all diets and, in addition, the HPM diet contained maize starch (pregelatinized, C-gel Instant, Cargill Nordic A/S, Charlottenlund, Denmark), sucrose (Danisco Sugar, Copenhagen, Denmark), crystalline cellulose (Macherey-Nagel GmbH & Co, Düren, Germany), vitamin/mineral mix (Brogaarden, Lynge, Denmark), L-cysteine (Sigma-Aldrich, Copenhagen, Denmark), and choline bitartrate (BDH Chemicals, Ltd., Poole, UK). For the other three experimental diets, 10% of the maize starch in the HPM was substituted by either 10% high-amylose maize starch (HAMS; High-maize^®^ 260, Ingredion, Bridgewater, NJ, USA), high-amylose potato starch (HAPS; KMC, Brande, Denmark), or butyrylated HAMS (HAMSB). The HAMSB was produced by an organocatalytic reaction with tartaric acid as a catalyst [24,25], resulting in a degree of substitution (DS) of approximately 0.5, measured by heterogenous saponification and back titration with HCl. The HAPS is a product of natural plant breeding and is not genetically modified. The ingredients of the four experimental diets and their chemical composition can be seen from Table 3.

All chemical analyses on diets were performed in duplicates, on freeze dried material. The dry matter (DM) content was determined by drying the samples at 103 °C, to constant weight, and ash was analyzed according to the AOAC method (923.03; AOAC) [49]. Nitrogen was measured by DUMAS [50] and protein was calculated as N × 6.25. Gross energy was determined with a LECO AC 300 automated calorimeter system 789–500 (LECO, St Joseph, MI, USA). Fat was determined using the Stoldt procedure [51], and starch was analyzed as described by Bach-Knudsen [52]. The analytical inaccuracies considered, the four diets were equal in protein (31–33%) and fat (19–21%). 

### 4.2. Animals

The care and housing of animals were in compliance with Danish laws and regulations for the humane care and use of animals in research (The Danish Ministry of Justice, Animal Testing Act, Consolidation Act No. 1306 of November 23, 2007) and was performed under the license obtained from the Danish Animal Experimentation Inspectorate, Ministry of Food, Agriculture, and Fisheries. The welfare of the animals was monitored and the rats stayed healthy throughout the experiment. 

A total of forty 6-week-old male Sprauge-Dawly rats (Taconic Europe, Ry, Denmark) with an initial body weight (BW) of 160 g were used for the experiment, conducted in three blocks of twelve rats in block 1 and 3, and sixteen rats in block 2 (3–4 rats per dietary treatment/block, in total *n* = 10 rats/diet). The rats were housed in pairs or three together, depending on the block, in standard cages, at constant temperature (25 °C), relative humidity (60%), and a 12:12-h light–dark cycle, with no natural light, and fed a standard rat chow (Altromin 1324, Brogaarden A/S, Gentofte, Denmark) ad libitum. After five days of acclimatization to the facility, rats were assigned to one of four experimental diets (Table 3), according to their BW and fed ad libitum for the following four weeks. BW was recorded once a week throughout the experiment.

Feed residues were weighed at cage level (2–3 rats/cage) and the average feed intake per rat per cage were calculated during week 1. At the end of the second week, rats were transferred to individual housing and ad libitum feeding in metabolic cages, allowing for separate collection of feces and urine [53]. Following the 3-day habituation period, feed residues were registered and feces collected quantitatively, every second day, for four days. After a total of 7 days in the metabolism cages, rats were transferred back to group housing (2–3 rats per cage) receiving the same diet as in the collection period, until final sampling and euthanasia, two days later.

### 4.3. Sampling and Analysis

Rats were anaesthetized with a mixture of Dormicum (5 mg/mL), Hypnorm, and sterile water (1:1:2; dose 0.3 mL/100 g). Blood (Vena Cava) was obtained and centrifuged for 10 min at 4 °C and 3,000 rpm and plasma was stored at −20 °C, until further analyzed. Following euthanasia by injection of pentobarbital into the heart, the cecum was weighed and digesta collected and stored at −20 °C, for further analysis. A 3 cm segment of the most distal end of the colon was fixed in 10% buffered formalin and dehydrated through gradient alcohol and xylene, before being embedded in paraffin wax. The following 4 cm segment of the distal end colon was gently rinsed and scraps of the intestinal mucosa were obtained with a glass cover slide. The mucosal scraps were placed in RNAlater, at 5 °C for 24 h before being stored at −80 °C, until further analyses.

Feces and cecum digesta were analyzed for short-chain fatty acids (SCFA) by gas chromatography (HP-6890 Series Gas Chromatograph, Hewlett Packard Palo Alto, CA), using an SGE-BP1 column (30 m × 0.25 mm × 0.25 µm; Trajan Scientific, Ringwood, Australia), with 5% phenylpolysiloxane and 95% dimethylpolysiloxane, and a flame ionization detector, after submitting the samples to an acid–base treatment, followed by ether extraction and derivatization [54]. The concentration of indole, phenol, and p-cresol in feces and the cecum digesta was measured by high-performance liquid chromatography (HPLC). The sample (0.5–1 g) was weighed in a blender bag and diluted 10-fold, with 0.028 M NaOH solution, containing 2.778 ppm indole-2-carboxylic acid as an internal standard (IS) and was homogenized for 2 min. The diluted sample (1 mL) was transferred to a glass centrifuge tube with a screw cap and 2 mL of HPLC grade methanol was added. The mixture was vortexed 30 s, placed at –80 °C for 5 min to accelerate the precipitation of the particulate matter, and centrifuged (3000× *g*, 10 min, 0 °C). Subsequently, 1 mL was transferred to a 1.5 mL micro tube and centrifuged (19,000× *g*, 10 min, 0 °C). A 150 µL aliquot was transferred to a 2 mL HPLC-vial containing 350 µL 50/50% *v*/*v* methanol/ultra-pure water and was analyzed in the HPLC system. The HPLC system was an Agilent 1200 series consisting of a vacuum degasser (G1379B), a binary pump (G1312A), an autosampler (G1329A), a thermostat for autosampler (G1330B), a thermostatted column compartment (G1316A), a fluorescence detector (G1321A), and a Chromeleon 7 software, for control of the HPLC system and data analyses. The column was a Phenomenex kinetic C18, 4.6 × 100 mm, 2.6 µm, fitted with a KrudKatcher ULTRA HPLC In-Line Filter (0.5 µm) and operated at 40 °C. The mobile phase consisted of (A) acetonitrile-50 mM potassium phosphate buffer (pH 6.0) 5:95% *v*/*v* and (B) acetonitrile-ultra pure water 90:10% v/v. The following gradient profile was used: 0–1 min, 5% B; 1–4 min, 5–40% B; 4–9 min, 40–52% B; 9–10 min, 52–100% B; 10–11 min, 100% B; 11–12 min, 100–5% B; 12–13 min, 5% B. The flowrate was 1.00 mL min^−1^. Temperature in the auto-sampler was set to 5 °C and 10 µL sample was injected in the system. Fluorescence detection was performed with excitation at 285 nm and emission at 340 nm. The standard mixture was prepared in methanol/ultra-pure water (50/50% *v*/*v*) and the concentration was as follows: IAA (0.5 ppm), IS (0.25 ppm), phenol (10.0 ppm), and p-cresol (2.5 ppm). Quantification of the compounds was based on peak area.

The pool size (µmol) of carbohydrate and protein fermentation products in cecum digesta and feces was calculated by multiplying the amount of cecum digesta or feces with the concentration in the wet digesta or feces.

### 4.4. Microbial Composition in the Cecum Digesta

Bacterial genomic DNA was extracted and the 16S rRNA gene V4 region was amplified for DNA sequencing, as previously described [55], using primers 515F and 806R, with some modifications. The number of PCR amplification cycles was decreased to 25, and the DNA in the pooled PCR amplicon library was purified by ethanol precipitation. Adapters were ligated to the amplicons at the UC Davis Genome Center (http://dnatech.genomecenter.ucdavis.edu/, accessed on: 19 June 2017)), with the KAPA Hyper Prep Kit, following the standard protocol. The 16S rRNA gene amplicons were sequenced, using a paired-end Illumina Mi-Seq (PE300) platform (Illumina Inc., San Diego, CA, USA) at the UC Davis Genome Center. 

DNA sequence analysis was performed using the pipeline Quantitative Insights Into Microbial Ecology (QIIME) version 2.8.0 [56]. The barcode regions were extracted with extract_barcodes.py, and demultiplexed using “qiime demux emp-single”. Only high-quality, forward-direction reads that met the following criteria were retained such that there were (i) no errors in the barcode, (ii) no ambiguous bases in the DNA sequence, and (iii) the sequence received at least the minimal acceptable Phred quality score of 30. Based on these criteria, a total of 3,547,758 high-quality reads were obtained, with an average of 88,027 ± 21,181 reads per sample. The DNA sequence reads were denoised with DADA2 [57], which inferred the amplicon sequence variants (ASVs). Representative sequences from each ASV were aligned to the reference Greengenes database (version gg-13-8-99-515-806,) [58] by the “qiime feature-classifier classify-sklearn” taxonomy assigner, and a phylogenetic tree was constructed. ASVs present in less than 5 samples or with a frequency of less than 10 total counts, were removed. These criteria yielded 2517 distinct ASVs and a total of 8 taxonomic levels in the dataset.

### 4.5. Oncogenic miRNA Expression Analysis

Total RNA was isolated using the mirVana™ miRNA Isolation Kit with phenol (Invitrogen, Fisher Scientific, Roskilde, Denmark), according to the supplier’s instructions. The isolated RNA samples were reverse transcribed, using the TaqMan^®^ MicroRNA Reverse Transcription Kit (Applied Biosystems, Fisher Scientific, Roskilde, Denmark), multiplexed with Taqman miRNA RT primers, specific for each miRNA of interest. Reverse transcription products were used for real-time quantitative PCR, in triplicates, using Taqman microRNA Assays (Applied Biosystems) with miRNA specific primers (Applied Biosystems assay IDs: miR-17-5p: 000393; miR-19a-3p: 000395; miR19b-3p: 000396; miR-20a-5p: 000580; miR-92-3p: 000430; miR-21-5p: 000397) and a TaqMan Fast Advanced Master Mix (Applied Biosystems), using the recommended PCR conditions on a ViiA 7 Real-Time PCR system (Applied Biosystems). The reference genes examined were β-actin (assay ID: Rn00667869-m1) and HPRT-1 (assay ID: Rn01527840-m1). The raw gene-expression data were obtained as Ct values, according to the manufacturer’s guidelines, and was used to determine the ΔCt values (ΔCt = Ct of the target gene - Ct of the reference gene), which were used for the statistical analyses. Then, ΔΔCt (= ΔCt for HPM diet −ΔCt, e.g., the HAPS diet) was calculated and the relative gene-expression was derived using the (1 + efficiencies)^−ΔΔCT^ method and the fold change (FC) was reported.

### 4.6. O^6^MeG DNA-Adduct Quantification

The level of O^6^MeG DNA-adducts in the distal colonic epithelial cells was quantified by immunohistochemistry, using an antibody for the O^6^MeG DNA adduct in the paraffin-embedded tissues, as described previously [47,59]. Sections (4 µm) of the tissue were rehydrated and antigen retrieval (10 mM citrate buffer) was performed in an EMS Antigen Retriever (Emgrid, Melbourne, Vic, Australia), followed by endogenous peroxidase blocking, by incubation in 3% H_2_O_2_ in water (10 min). RNase treatment was then performed (RNase A 400 U/mL + RNase T 10 U/mL); PBS (pH 7.4) in a humidity chamber (37 °C, 15 min) and stopped with NaCl solution (140 mM, at 4 °C, 15 min). Alkali treatment was performed (4 °C, 5 min) to unwind the DNA and expose the sites of interest. Following two times washing with Tris-buffered saline (TBS), the Serum Block reagent (Covance Laboratories, Princeton, NJ, USA) was applied (room temp, 30 min). The O^6^ MeG antibody (1:250; Clone EM 2–3; Squarix Biotechnology, Marl, Germany) was applied to the slides overnight (4 °C), followed by a “Boost” reagent (Covance; room temp, 30 min), before applying the poly-horseradish peroxidase (HRP) anti- mouse IgG (Covance). Positive cells were visualized through the addition of 3,3´-diaminobenzamine tetrahydrochloride (DAB) chromogen (Covance), and sections were counterstained with hematoxylin. A light microscope (Olympus BX41, Olympus Australia Pty Ltd, Notting Hill, Victoria, Australia) was used for the observation of slides, with an image capture of the entire crypt lengths being undertaken, using a Q Imaging Micropublisher 3.3 RTV digital camera and an Olysia Bioreport Imaging System 5.0 software. Microscope and camera settings remained the same for each image captured. The program R for Windows 3.3.3 and Q capture suite 2.68.6.0 were used to assess the images. The ratio of positive “red” staining for the O^6^MeG adduct to the negative “blue” staining for the individual cell nuclei (RoB ratio), was determined. A higher RoB ratio indicated a greater O^6^MeG staining intensity, correlating to a higher O^6^MeG adduct load. Each cell along the entire crypt length was measured for the RoB ratio, and 20 crypts per rat were scored.

### 4.7. Statistical Analysis

Data on the fermentation products, the average staining intensity of the colonic crypts (O^6^MeG adduct formation) and colonic miRNA expression was analyzed, using the Mixed procedure of SAS (SAS institute, Inc., Cary, NC, USA), according to the following ANOVA model:

$$X_{\left(ij\right)}=\;µ\;+\alpha_{\left(i\right)}+\upsilon_{\left(j\right)}+{\alpha \upsilon }_{\left(ij\right)}+\varepsilon_{\left(ij\right)}$$

where α_(*i*)_ is the diet (*i* = HPM, HAPS, HAMS or HAMSB); υ_(*j*)_ is the random effect of block (*j* = 1, 2 or 3); αυ_(*ij*)_ is the interaction between the diet and the block, and ε_(*ij*)_ denotes the residual error. Levels of significance was reported as being significant when *p* ≤ 0.05. The random effect and residuals were assumed to be normally distributed and independent and their expectations were assumed to be zero. 

In the analysis of the cecal microbial composition, alpha diversity was quantified using the R packages Phyloseq and Picante [60,61]. Beta diversity was calculated using weighted UniFrac distances and constrained correspondence analysis for ordination. Statistical differences for both alpha and beta diversity were assessed by ANOVA, followed by the post hoc Tukey HSD test in R. To calculate abundance, Phyloseq was used to transform the sample counts of the Phyloseq object, followed by agglomeration to the indicated taxonomic level, and plotting with the R package ggplot [60]. Differentially abundant taxa were calculated using DESeq2 [62]; an adjusted *p*-value < 0.05, log_2_ changes greater than ±1, and a mean relative abundance ≥ 0.005, in at least one rat dietary group were considered to be significant. The mean relative abundance was calculated in Phyloseq by transforming the counts to the relative abundance, then agglomerating to the indicated level [60].

## Figures and Tables

**Table 1:** Selected carbohydrate and protein fermentation products in the cecum digesta and feces of rats fed the four different diets (high-protein meat, HPM; high-amylose potato starch, HAPS; high-amylose maize starch, HAMS; butyrylated high-amylose maize starch, HAMSB). Least square mean values with their standard errors, *n* = 10 per group.

	HPM	HAPS	HAMS	HAMSB	SEM	*p*
**Cecum, concentration**						
Acetate, µmol/g	51.0 ^b^	60.4 ^a^	61.7 ^a^	47.7 ^b^	3.6	<0.01
Propionate, µmol/g	11.2	11.5	10.2	9.8	0.6	0.22
Butyrate, µmol/g	12.1 ^d^	18.9 ^b^	15.1 ^c^	23.9 ^a^	1.5	<0.001
Total org. acids ^1^, µmol/g	79.8 ^c^	97.5 ^a^	92.3 ^ab^	86.3 ^bc^	4.1	0.03
BCFA ^2^, µmol/g	2.2 ^a^	1.9 ^a^	1.5 ^b^	1.0 ^c^	0.1	<0.001
p-cresol, µg/g	14.4 ^a^	10.0 ^ab^	7.7 ^b^	6.5 ^b^	1.7	0.02
Phenol, µg/g	8.1 ^a^	6.9 ^a^	1.6 ^b^	5.5 ^a^	1.4	0.01
Indole, µg/g	19.7 ^ab^	20.9 ^a^	13.5 ^c^	16.0 ^bc^	1.5	<0.01
**Cecum, pool**						
Cecum digesta, g, wet weight	1.8 ^b^	1.9 ^b^	2.4 ^a^	2.6 ^a^	0.1	<0.001
Acetate, µmol	89 ^c^	113 ^b^	156 ^a^	125 ^b^	7	<0.001
Propionate, µmol	20	21	24	26	2	0.12
Butyrate, µmol	21 ^c^	35 ^b^	36 ^b^	62 ^a^	3	<0.001
Total org. acids ^1^, µmol	139 ^c^	181 ^b^	233 ^a^	226 ^a^	11	<0.001
BCFA ^2^, µmol	3.8 ^a^	3.7 ^a^	3.4 ^ab^	2.5 ^b^	0.4	0.05
p-cresol, µg	26	18	17	17	3	0.19
Phenol, µg	14 ^a^	18 ^a^	4 ^b^	16 ^a^	3	0.03
Indole, µg	34	39	32	42	4	0.23
**Feces, concentration**						
Acetate, µmol/g	31.7 ^b^	38.3 ^b^	61.3 ^a^	68.6 ^a^	4.8	<0.001
Propionate, µmol/g	3.4 ^b^	3.1 ^b^	3.7 ^b^	7.7 ^a^	0.8	<0.001
Butyrate, µmol/g ^4^	0.78 ^b^ [0.51;1.19]	0.73 ^b^ [0.48;1.09]	1.1 ^b^ [0.78;1.67]	16.0 ^a^ [0.48;1.09]		<0.001
Total org. acids ^1^, µmol/g	43.5 ^b^	51.3 ^b^	138.4 ^a^	115.0 ^a^	8.5	<0.001
BCFA ^1^, µmol/g ^4^	0.94 ^ab^ [0.68;1.28]	1.40 ^a^ [1.02;1.92]	0.60 ^bc^ [0.39;0.91]	0.54 ^c^ [0.39;0.76]		<0.01
p-cresol, µg/g	25.7 ^a^	18.8 ^b^	5.2 ^c^	15.1 ^b^	1.6	<0.001
Phenol, µg/g ^5^	4.3	3.6	0.0	0.59	1.4	0.08
Indole, µg/g	19.2 ^a^	14.3 ^b^	1.5 ^d^	8.8 ^c^	0.8	<0.001
**Feces pool**						
Feces ^3^, g wet weight/d	2.3 ^c^	2.8 ^c^	3.4 ^b^	4.8 ^a^	0.1	<0.001
Acetate, µmol	73 ^c^	103 ^c^	206 ^b^	329 ^a^	24	<0.001
Propionate, µmol ^4^	7 ^b^ [5.4;9.9]	9 ^b^ [6.3;11.5]	12 ^b^ [8.6;15.6]	40 ^a^ [27.4;50.0]		<0.001
Butyrate, µmol ^4^	2 ^b^ [1.2;2.7]	2 ^b^ [1.4;3.0]	4 ^b^ [2.6;5.4]	64 ^a^ [43.3;93.2]		<0.001
Total org. acids, µmol	101 ^b^	137 ^b^	448 ^a^	547 ^a^	42	<0.001
BCFA ^1^, µmol ^4^	2.2 [1.52;3.05]	3.8 [2.69;5.38]	1.9 [1.21;3.09]	2.5 [1.74;3.60]		0.08
p-cresol, µg	60 ^a^	50 ^a^	17 ^b^	64 ^a^	5	<0.001
Phenol, µg ^5^	16	10	0	3	5	0.16
Indole, µg	44 ^a^	39 ^a^	5 ^b^	41 ^a^	3	<0.001

^a,b,c,d^ Mean values within a row with unlike superscript letters were significantly different (*p* < 0.05). ^1^ Total organic acids = sum of formate, acetate, propionate, burtyrate, isobutyrate, isovalerate, isocaproate, lactate, and succinate ^2^ Branched-chain fatty acids (BCFA) = sum of iso-butyrate and iso-valerate. ^3^ Wet weight calculated over a five day period during week 3 (individual housing in metabolism cages). ^4^ Presented as least squares (LS)means ± 95% confidence intervals, due to logarithmic transformation of data. ^5^ Only 10 of the 40 observations were over the detection limit for phenol.

**Table 2:** Bacterial proportions that differed between rats in a diet-dependent manner.

	HPM	HAPS	HAMS	HAMSB
*o_Bifidobacteriales*	0.001 ± 0.0001 ^1a^	**0.017 ± 0.036 ^b^**	0.0018 ± 0.003 ^bc^	0.001 ± 0.0001 ^a^
*g_Parabacteroides*	0.012 ± 0.011 ^a^	0.003 ± 0.003 ^c^	0.006 ± 0.005 ^ac^	**0.078 ± 0.099 ^b^**
*f_S24-7*	0.033 ± 0.01 ^a^	**0.052 ± 0.027 ^b^**	0.040 ± 0.018 ^ab^	0.027 ± 0.012 ^a^
*f_Turicibacteraceae*	0.0001 ± 0.0001 ^a^	**0.0070 ± 0.012 ^b^**	0.0004 ± 0.0007 ^c^	0.0020 ± 0.003 ^bc^
*f_Peptostreptococcaceae*	**0.037 ± 0.028 ^a^**	0.014 ± 0.014 ^ab^	0.010 ± 0.008 ^b^	0.013 ± 0.009 ^ab^
*Ruminococcus bromii*	0.010 ± 0.005 ^a^	0.018 ± 0.014 ^a^	**0.278 ± 0.123 ^b^**	0.005 ± 0.001 ^a^
*f_Lactobacillaceae*	0.215 ± 0.0589 ^ab^	**0.263 ± 0.127 ^a^**	0.111 ± 0.075 ^b^	0.142 ± 0.054 ^b^
*g_Bilophila*	**0.013 ± 0.005 ^a^**	0.007 ± 0.006 ^ab^	0.008 ± 0.006 ^ab^	0.003 ± 0.005 ^b^
*g_Sutterella*	**0.008 ± 0.003 ^a^**	0.003 ± 0.003 ^b^	0.007 ± 0.007 ^ab^	0.004 ± 0.008 ^b^

^1^ Proportions of total 16S rRNA gene reads and their standard deviations are presented. For each taxon, the highest proportion found among the four diets is highlighted in bold. ^a,b,c^ Unlike superscript letters indicate significant differences (*p* < 0.05) in taxon abundance. DESeq2 was used to calculate the change in abundance using the Wald test, retaining only *p* < 0.05 and a log_2_ fold-change greater than ±1.

**Table 3:** Ingredients and analyzed chemical composition of the four experimental diets.

	Diet			
	HPM	HAPS	HAMS	HAMSB
**Ingredient (g/kg, as-fed basis)**				
Maize starch	283	183	183	183
Sugar	100	100	100	100
Cellulose	50	50	50	50
AIN 93G Mineral Mix	35	35	35	35
AIN 93G Vitamin Mix	10	10	10	10
L-cysteine	3.0	3.0	3.0	3.0
Choline Bitartrate	2.5	2.5	2.5	2.5
t-Butylhydroquinone	0.14	0.14	0.14	0.14
Beef ^1^	517	517	517	517
High-amylose potato starch (HAPS) ^2^		100		
High-amylose maize starch (HAMS) ^3^			100	
HAMS-butyrylated (HAMSB) ^4^				100
**Chemical composition (% of dry matter, DM)**				
DM (%)	96.5	96.6	96.9	96.6
Protein (N × 6.25)	32.5	31.2	32.6	30.8
Starch	30.9	23.9	25.3	26.8
Fat	20.8	19.0	19.1	18.6
Ash	3.9	4.1	4.0	4.2

^1^ Beef: Minced low-fat beef, cooked, dried (45 °C for 48 h) and milled (1 mm screen). ^2^ Amylose constituted 79.6% of DM (Megazyme amylose/amylopectin kit, Megazyme International, Wicklow, Ireland) and RS constituted 25.3% of DM (Megazyme RS kit, Megazyme International, Wicklow, Ireland). ^3^ Amylose constituted 41.5% of DM (Megazyme amylose/amylopectin kit, Megazyme International, Wicklow, Ireland) and RS constituted 39.9% of DM (Megazyme RS kit, Megazyme International, Wicklow, Ireland). ^4^ The RS content of HAMSB was not measured but assumed to be similar to HAMS or higher, due to esterification with butyrate

## Supplementary Materials

Supplementary materials can be found at https://www.mdpi.com/1422-0067/20/9/2137/s1.
