# Supplementary material for: High-Amylose Maize, Potato, and Butyrylated Starch Modulate Large Intestinal Fermentation, Microbial Composition, and Oncogenic miRNA Expression in Rats Fed A High-Protein Meat Diet
Source: Int J Mol Sci. 2019 Apr 30;20(9):2137. doi: 10.3390/ijms20092137 (PMC6540251; doi:10.3390/ijms20092137)
Supplement: Supplementary file 1 [file ijms-20-02137-s001.pdf]

Supplemental Figure

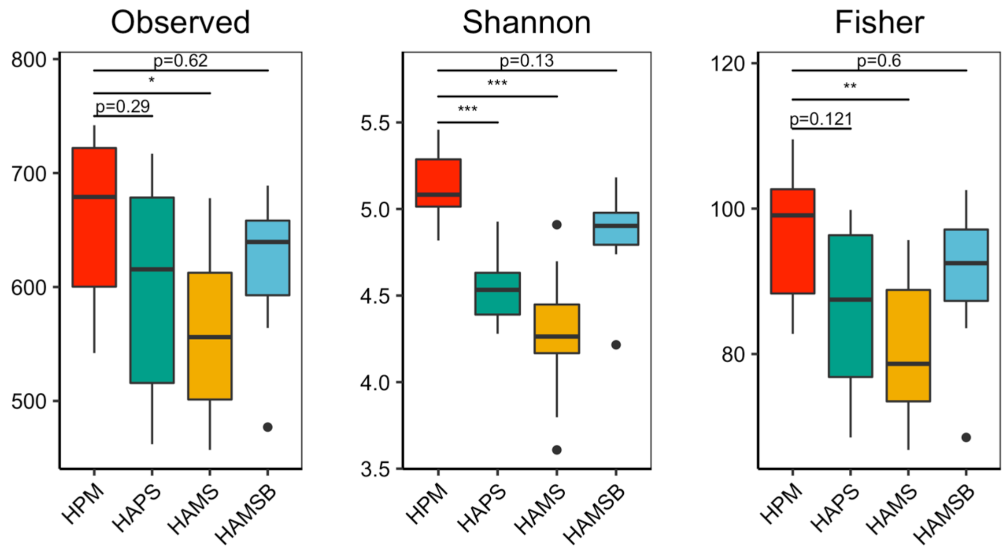

Suppl. Figure S1. Alpha diversity metrics of the rat caecal microbiota. (A) Observed OTU's (*p*=0.0439), (B) Shannon index (*p*=2.33e-07), and (C) Fisher's alpha test are shown (*p*=0.00965). ANOVA followed by Tukeys HSD was used to determine statistical significance (* $p < 0.05$, ** $p < 0.01$, *** $p < 0.001$). HPM: high protein meat, HAPS: HPM + high-amylose potato starch, HAMS: HPM + high amylose maize starch, HAMSB: HPM + butyrylated high-amylose maize starch.
